# Coenzyme Q_10_ supplementation improves metabolic parameters, liver function and mitochondrial respiration in rats with high doses of atorvastatin and a cholesterol-rich diet

**DOI:** 10.1186/1476-511X-13-22

**Published:** 2014-01-25

**Authors:** Ma Antonia Jiménez-Santos, Isela E Juárez-Rojop, Carlos A Tovilla-Zárate, María Teresa Espinosa-García, Marco A Juárez-Oropeza, Teresa Ramón-Frías, Deysi Y Bermúdez-Ocaña, Juan C Díaz-Zagoya

**Affiliations:** 1 División Académica Multidisciplinaria de Comalcalco, Universidad Juárez Autónoma de Tabasco, Comalcalco, Tabasco, México; 2 Centro de Investigación, DACS, Universidad Juárez Autónoma de Tabasco (UJAT), Villahermosa, Tabasco 86150, México; 3 Departamento de Bioquímica, Facultad de Medicina, UNAM, México, D.F, México

**Keywords:** Coenzyme Q_10_, Atorvastatin, Hypercholesterolemia

## Abstract

**Background:**

The aim of this study was to evaluate the actions of coenzyme Q_10_ (CoQ_10_) on rats with a cholesterol-rich diet (HD) and high doses of atorvastatin (ATV, 0.2, 0.56 or 1.42 mg/day).

**Methods:**

Two experiments were done, the first one without coenzyme Q_10_ supplementation. On the second experiment all groups received coenzyme Q_10_ 0.57 mg/day as supplement. After a 6-week treatment animals were sacrificed, blood and liver were analyzed and liver mitochondria were isolated and its oxygen consumption was evaluated in state 3 (phosphorylating state) and state 4 (resting state) in order to calculate the respiratory control (RC).

**Results:**

HD increased serum and hepatic cholesterol levels in rats with or without CoQ_10_. ATV reduced these values but CoQ_10_ improved even more serum and liver cholesterol. Triacylglycerols (TAG) were also lower in blood and liver of rats with ATV + CoQ_10_. HDL-C decreased in HD rats. Treatment with ATV maintained HDL-C levels. However, these values were lower in HD + CoQ_10_ compared to control diet (CD) + CoQ_10_. RC was lessened in liver mitochondria of HD. The administration of ATV increased RC. All groups supplemented with CoQ_10_ showed an increment in RC. In conclusion, the combined administration of ATV and CoQ_10_ improved biochemical parameters, liver function and mitochondrial respiration in hypercholesterolemic rats.

**Conclusions:**

Our results suggest a potential beneficial effect of CoQ_10_ supplementation in hypercholesterolemic rats that also receive atorvastatin. This beneficial effect of CoQ_10_ must be combined with statin treatment in patient with high levels of cholesterol.

## Background

Hypercholesterolemia is considered a risk factor for atherosclerosis and cardiovascular disease. The World Health Organization expectancy for 2020 is a death rate of 71% due to ischemic cardiomyopathy [1,2]. According to the 2012 National Health Survey [3], the prevalence of hypercholesterolemia in Mexico was 13% in adult population. As it is well known, statins constitute the current therapeutic tool for hyperlipidemia. Atorvastatin is widely used by clinicians due to its competitive action on HMG-CoA reductase that results in a decrement of plasma total cholesterol, low density lipoprotein cholesterol (LDL-C) and very low density lipoprotein cholesterol (VLDL-C). It also reduces apolipoprotein B and the triacylglycerol levels [4]. The hypocholesterolemic action of statins is well known in human beings and in animal models. Statins also produce lower levels of plasma cholesterol and triacylglycerol, and higher levels of high density lipoprotein cholesterol (HDL-C) [5]. Statins are generally well tolerated, however they may show undesirable effects such as myositis, rabdomyolysis and liver damage, but their beneficial actions exceed their collateral effects and so they continue being the first choice for the prevention of coronary cardiovascular disease [6].

Mevalonate is a precursor of endogenous cholesterol and other metabolites like ubiquinone, dolichol and other isoprenoids [7]. Ubiquinone is also known as coenzyme Q_10_ (CoQ_10_), it belongs to a family of compounds that share a common structure, the benzoquinone ring, and differ in their isoprenoid lateral chain length; CoQ_10_ is a redox component of the mitochondrial respiratory chain that synthesizes ATP. The reduced form of CoQ_10_ (ubiquinol) is a powerful lipophilic antioxidant that participates in tocopherol and ascorbate recycling as antioxidants [8]. Other reports suggest that statins reduce CoQ_10_ biosynthesis in the liver. This reduced content could diminish oxygen consumption by the mitochondria and therefore affect the respiratory control. The aim of this study was to evaluate the role of CoQ_10_ on metabolic parameters, liver function and mitochondrial respiration in rats with high doses of atorvastatin and a cholesterol-rich diet, a condition which is severely harmful [9] in rodents. It was employed a ATV_1_ (0.2 mg/day) dose in rats (200 g body weight) because it is equivalent to a ATV dose of 60 mg/day in a human being (60 Kg body weight). ATV_2_ (0.56 mg/day) and ATV_3_ (1.42 mg/day) correspond to 2.8 and 7 fold higher doses.

## Results

### Body weight gain and liver weight as a percentage of body weight

The HD rats showed a higher body weight gain compared to CD rats (p < 0.05), however, there were not significant differences in groups HD + ATV_1, 2, 3_ compared with HD without CoQ_10_. HD + ATV_1_ + CoQ_10_ showed less weight gain (77 ± 28 g) with respect to HD + CoQ_10_ (p < 0.05) (Table 1). A similar pattern was observed between HD + ATV_3_ + CoQ_10_ and HD + CoQ_10_ (p < 0.05). Moreover, there was an important diminution of body weight gain in all groups that received CoQ_10_ supplementation in comparison with the same groups without CoQ_10_ (p < 0.05). On the other hand, the liver percentage relative to body weight showed a significant decrement in CD + ATV_2_ (2.7 ± 0.1%) compared to CD (2.9 ± 0.2%), and a significantly increment in HD (4.7 ± 1.3%) also in comparison with CD (p < 0.05). Besides, HD + ATV_2_ + CoQ_10_ (3.08 ± 0.13%) and HD + ATV_3_ + CoQ_10_ (3.2 ± 0.18%) showed a significant decrement relative to HD + CoQ_10_ (3.9 ± 1.1%). The weight gain and the liver percentage relative to body weight were significantly different between the respective groups with and without supplementation of CoQ_10_ in their diet.

**Table 1:** **Weight gain and liver percentage relative to body weight in rats with cholesterol**-**rich diet**, **atorvastatin and with or without coenzyme Q**_**10 **_**supplementation**

	**CD**	**CD + ATV**_**2 **_**(0.56 /day)**	**HD**	**HD + ATV**_**1 **_**(0.2 g/day)**	**HD + ATV**_**2 **_**(0.56 mg/day)**	**HD + ATV**_**3 **_**(1.42 mg/day)**
Body weight gain (g)						
Without CoQ_10_	114 ± 20	126 ± 23	140 ± 4ª	130 ± 7	135 ± 24	104 ± 38
With CoQ_10_	95 ± 7^1^	102 ± 36^1^	117 ± 8^a,1^	77 ± 28^b,1^	108 ± 6^1^	96 ± 8^b,1^
Liver percentage						
Relative to body weight (%)						
Without CoQ_10_	2.9 ± 0.2	2.7 ± 0.1^a^	4.7 ± 1.3^a^	4.5 ± 0.3	4.3 ± 0.3	4.3 ± 0.5
With CoQ_10_	2.38 ± 0.1^1^	3.2 ± 0.1^a, 1^	3.92 ± 1.1^a^	3.9 ± 0.1^1^	3.08 ± 0.13^b, 1^	3.2 ± 0.18^b, 1^

CD, control diet; CD + ATV_2_, CD + atorvastatin 0.56 mg/day; HD, hypercholesterolemic diet; HD + ATV_1_, 0.2 mg/day; HD + ATV_2_ 0.56 mg/day; HD + ATV_3_ 1.42 mg/day, Coenzyme Q_10_, 0.57 mg/day; Data are expressed as mean ± S.E.M.; n = 8. One-way analysis of variance (ANOVA) (groups CD, CD + ATV_2_, HD, HD + ATV_1_, HD + ATV_2_, HD + ATV_3_) accompanied by the Student-Newman-Keuls test and differences between the groups were determined by the Student's t test (Without CoQ_10_ vs With CoQ_10_) (p < 0.05). ^a^Statistically different from CD (p < 0.05); ^b^statistically different from either HD or HD + CoQ_10_ (p < 0.05); ^1^statistically different from the same group without CoQ_10_.

### Biochemical parameters

**Figure 1:**
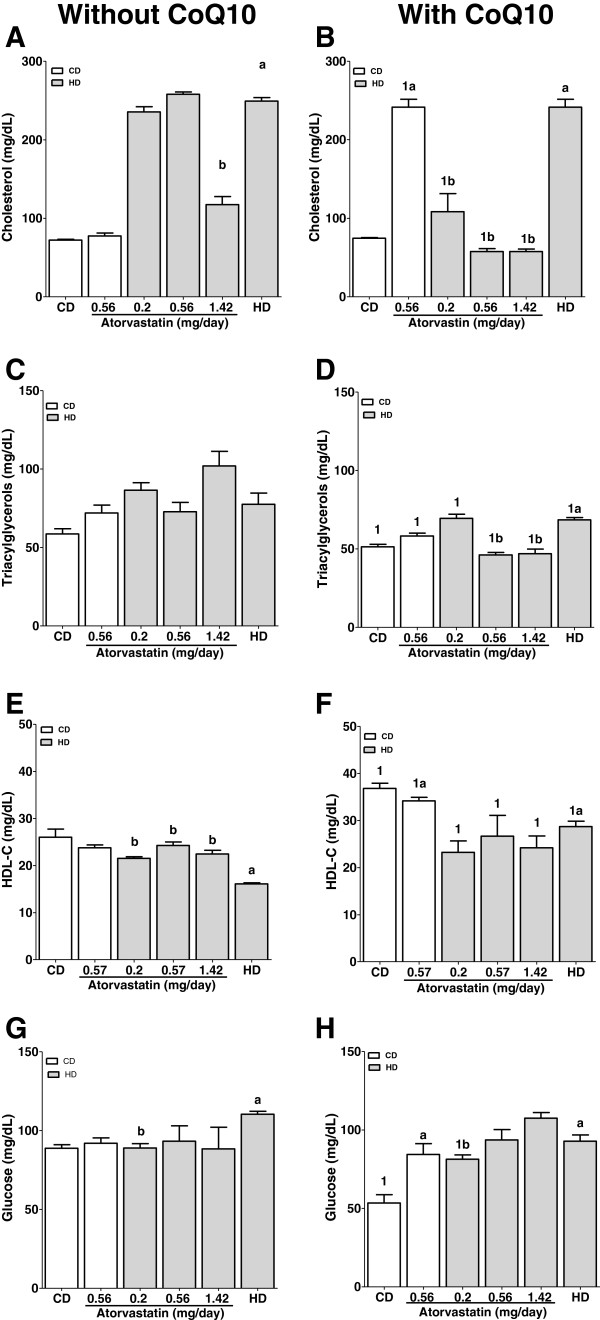
**Effect of a cholesterol**-**rich diet and atorvastatin given to rats with and without coenzyme Q**_**10 **_**supplementation on biochemical parameters.** Each bar is the mean ± S.E.M. of eight animals. *P < 0.05. Statistical analysis was done by One-way analysis of variance (ANOVA), followed by the Student-Newman–Keuls test and differences between the groups were determined by the Student's t test (Without CoQ_10_ vs With CoQ_10_). ^a^Statistically different from CD; ^b^statistically different from HD; ^1^statistically different from the same group without CoQ_10_ (p < 0.05). **A**, **C**, **E**, **G** without CoQ_10_; **B**, **D**, **F**, **H** with Q_10_. HDL-C (High density lipoprotein-Cholesterol).

The administration of a cholesterol-rich diet produced elevated levels of plasma cholesterol in rats with or without CoQ_10_ supplementation (249.3 ± 9.0 mg/dL, and 241.5 ± 26.4 mg/dL, respectively) compared with CD (72.2 ± 2.49 mg/dL) and with CD + ATV_2_ (77.6 ± 3.6 mg/dL); on the other hand, the supplementation with CoQ_10_ produced significant lower values of serum cholesterol in HD + ATV_1, 2, 3_ + CoQ_10_ compared with the same groups without CoQ_10_ (p < 0.05) (Figure 1A, B).

Triacylglycerol concentration in serum increased in HD (77.5 ± 12.5 mg/dL) in comparison with CD (58.6 ± 7.4 mg/dL). HD + ATV_1, 2, 3_ did not diminish the TAG levels in comparison with HD, however, in HD + ATV_3_, TAG levels were higher (102 ± 16 mg/dL) in comparison with HD (77.5 ± 12.5 mg/dL). All TAG values were minor when CoQ_10_ was added as a supplement (Figure 1C, D) (p < 0.05).

HDL-C in serum showed a significant decrease in HD (16.1 ± 0.4 mg/dL) with respect to CD (26.0 ± 2.6 mg/dL) (p < 0.05). Treatment with HD + ATV_1, 2, 3_ maintained HDL-C levels similar to those of CD group, but lower to those of CD + CoQ10 (36.82 ± 2.7 mg/dL). Groups CD + CoQ_10_, HD + CoQ_10_ and CD + ATV_2_ + CoQ_10_ showed higher values (36.8 ± 2.7 mg/dL, 28.7 ± 3.2 mg/dL, 34.2 ± 2.1 mg/dL, respectively) than CD without CoQ_10_ (26.0 ± 2.6 mg/dL). However, HDL-C levels were lower in HD + ATV_1, 2, 3,_ + CoQ_10_ (20.2 ± 1.5, 18.6 ± 0.6, 21.8 ± 1.4 mg/dL, respectively) than in CD + CoQ_10_ (36.82 ± 2.7 mg/dL) (Figure 1E, F).

Serum glucose levels significantly increased in HD and HD + CoQ_10_ (110.2 ± 1.2 mg/dL and 99.6 ± 0.7 mg/dL, respectively) in comparison with CD and CD + CoQ_10_ (88.8 ± 4.9 mg/dL; 53.4 ± 12.9, respectively). The treatment with HD + ATV_1_ decreased glucose level. However, it was obtained a significant diminution in groups CD, CD + ATV_2_, HD and HD + ATV_1_ treated with CoQ_10_ supplementation (Figure 1G, H) in comparison with HD not supplemented.

HD administration induced a slight increase in serum activity of ALT and AST (84.8 ± 7.9 and 223.6 ± 14.27 IU/L, respectively) in comparison with CD (67.1 ± 9.19 and 165.2 ± 9.7 IU/L, respectively). ATV administration produced a significant decrease in ALT activity in HD + ATV_2_ (60.9 ± 2.5 IU/L) and HD + ATV_3_ (57.1 ± 5.1 IU/L) in comparison with HD (84.8 ± 7.9 IU/L) (p <0, 05). CoQ_10_ supplementation decreased ALT activity in all groups with cholesterol-rich diet and ATV (Table 2). On the other hand, serum AST activity increased in CD + ATV_2_ (209.2 ± 10.8 IU/L) compared with CD (165.2 ± 9.7 IU/L) and increased in HD + ATV_1_ (273.7 ± 6.98 IU/L) in comparison with HD (223.6 ± 14.27 IU/L) (p <0, 05).

**Table 2:** **Effect of a cholesterol**-**rich diet and atorvastatin with or without coenzyme Q**_**10 **_**supplementation on transaminases**

	**CD**	**CD + ATV**_**2**_	**HD**	**HD + ATV**_**1**_	**HD + ATV**_**2**_	**HD + ATV**_**3**_
		**(0.56 mg/day)**		**(0.2 mg/day)**	**(0.56 mg/day)**	**(1.42 mg/day)**
ALT						
Without CoQ_10_	67.1 ± 9.19	56.32 ± 0.8^a^	84.8 ± 7.9^a^	128.0 ± 8.9^b^	60.9 ± 2.5^b^	57.10 ± 5.11^b^
With CoQ_10_	49.05 ± 5.58	48.33 ± 3.86^1^	102.6 ± 9.9^1^	94.10 ± 18.40	44.15 ± 6.45^b,1^	56.8 ± 0.75^b^
AST						
Without CoQ_10_	165.2 ± 9.7	209.2 ± 10.83^a^	223.6 ± 14.27^a^	273.7 ± 6.98^b^	224.6 ± 8.8	196.0 ± 7.31^b^
With CoQ_10_	227.8 ± 1.98	164.0 ± 1.90^a,1^	313.3 ± 4.87^a,1^	239.9 ± 9.05^b,1^	261.4 ± 11.20^b,1^	247.5 ± 15.45^b,1^

CD, control diet; CD + ATV_2_, CD + atorvastatin 0.56 mg/day; HD, hypercholesterolemic diet; HD + ATV_1_, 0.2 mg/day; HD + ATV_2_ 0.56 mg/day; HD + ATV_3_ 1.42 mg/day, Coenzyme Q_10_, 0.57 mg/day; Data are expressed as mean ± S.E.M.; n = 8. One-way analysis of variance (ANOVA) (groups CD, CD + ATV_2_, HD, HD + ATV_1_, HD + ATV_2_, HD + ATV_3_) accompanied by the Student-Newman-Keuls test and differences between the groups were determined by the Student's t test (Without CoQ10 vs With CoQ10) (p < 0.05). ^a^Statistically different from either CD or CD + CoQ_10_ (p < 0.05); ^b^statistically different from either HD or HD + CoQ_10_ (p < 0.05); ^1^statistically different from the same group without CoQ_10_.

### Cholesterol and triacylglycerols from the liver

The administration of a cholesterol-rich diet produced a significant increase in hepatic cholesterol in HD (12.43 ± 2.84 mg/g) and HD + CoQ_10_ (6.42 ± 0.86 mg/g) compared with CD (2.71 ± 0.86 mg/g) or CD + CoQ_10_ (2.30 ± 0.75 mg/g). ATV administration in HD + ATV_1,2,3_ decreased cholesterol levels in comparison with HD and a more significant response was obtained when CoQ_10_ was supplemented.

Liver triacylglycerol levels were also increased in HD (10.66 ± 0.33 mg/g) and HD + CoQ_10_ (10.7 ± 0.32 mg/g) in comparison with CD (5.58 ± 0.88 mg/g) and CD + CoQ_10_ (4.30 ± 0.55 mg/g). On the other hand, HD + ATV_1, 2, 3_ diminished TAG levels when compared with HD, but only with the highest dose the difference was significant. All the groups HD + ATV with CoQ_10_ supplementation showed lower values in hepatic TAG levels than those observed in HD + CoQ_10_ group (Table 3).

**Table 3:** **Effect of CoQ**_**10 **_**supplementation on liver cholesterol and triacylglycerols of rats with a cholesterol**-**rich diet and atorvastatin**

	**CD**	**CD + ATV**_**2**_	**HD**	**HD + ATV**_**1**_	**HD + ATV**_**2**_	**HD + ATV**_**3**_
		**(0.56 mg/day)**		**(0.2 mg/day)**	**(0.56 mg/day)**	**(1.42 mg/day)**
TC						
Without CoQ_10_	2.71 ± 0.86	2.77 ± 0.33	12.43 ± 2.84^a^	7.80 ± 1.73^b^	8.71 ± 0.62^b^	7.38 ± 1.15^b^
With CoQ_10_	2.30 ± 0.75	1.87 ± 0.34^a,1^	6.42 ± 0.86^a,1^	2.42 ± 0.47^b,1^	2.25 ± 0.46^b,1^	3.30 ± 0.75^b,1^
TAG						
Without CoQ_10_	5.58 ± 0.88	7.53 ± 0.45^a^	10.66 ± 0.33^a^	9.73 ± 1.95	9.70 ± 1.50	6.31 ± 2.23^b^
With CoQ_10_	4.30 ± 0.55^1^	6.24 ± 1.16^a,1^	10.70 ± 0.32^a^	3.73 ± 0.60^b,1^	2.81 ± 0.11^b, 1^	6.47 ± 0.20^b^

CD, control diet; CD + ATV_2_, CD + atorvastatin 0.56 mg/day; HD, hypercholesterolemic diet; HD + ATV_1_, 0.2 mg/day; HD + ATV_2_ 0.56 mg/day; HD + ATV_3_ 1.42 mg/day, Coenzyme Q_10_, 0.57 mg/day; Data are expressed as mean ± S.E.M.; n = 8. One-way analysis of variance (ANOVA) (groups CD, CD + ATV_2_, HD, HD + ATV_1_, HD + ATV_2_, HD + ATV_3_) accompanied by the Student-Newman-Keuls test and differences between the groups were determined by the Student's t test (Without CoQ_10_ vs With CoQ_10_) (p < 0.05). ^a^Statistically different from either CD or CD + CoQ_10_ (p < 0.05); ^b^statistically different from either HD or HD + CoQ_10_ (p < 0.05); ^1^statistically different from the same group without CoQ_10_.

### Respiratory control

The respiratory control (RC) was lessened in the liver mitochondria of HD rats (2.02 ± 0.5) in comparison with CD (2.98 ± 0.06). The treatment of ATV_1_ or ATV_3_ to HD rats induced a significant increase in oxygen consumption (2.93 ± 0.3 and 2.38 ± 0.35, respectively) in comparison to HD (2.02 ± 0.5). However, HD + ATV_2_ showed a lower RC (1.85 ± 0.15) in comparison to HD (2.02 ± 0.5). All groups but HD + ATV_1_ showed an increment in RC when they were supplemented with CoQ_10_ (p < 0.05) in comparison with the same groups without CoQ_10_ supplementation (Table 4).

**Table 4:** **Effect of a cholesterol**-**rich diet and atorvastatin with or without coenzyme Q**_**10 **_**supplementation on the liver mitochondrial respiratory control index**

**Respiratory control**	**CD**	**CD + ATV**_**2**_	**HD**	**HD + ATV**_**1**_	**HD + ATV**_**2**_	**HD + ATV**_**3**_
		**(0.56 mg/day)**		**(0.2 mg/day)**	**(0.56 g/day)**	**(1.42 mg/day)**
Without CoQ_10_	2.98 ± 0.06	2.25 ± 0.25^a^	2.02 ± 0.51^a^	2.93 ± 0.27^b^	1.85 ± 0.15	2.38 ± 0.35
With CoQ_10_	3.20 ± 0.15^1^	4.69 ± 0.27^a,1^	3.68 ± 0.21^a,1^	2.72 ± 0.40^b^	3.55 ± 0.02^1^	3.13 ± 0.22^1^

CD, control diet; CD + ATV_2_, CD + atorvastatin 0.56 mg/day; HD, hypercholesterolemic diet; HD + ATV_1_, 0.2 mg/day; HD + ATV2 0.56 mg/day; HD + ATV_3_ 1.42 mg/day, Coenzyme Q_10_, 0.57 mg/day; Data are expressed as mean ± S.E.M.; n = 8. One-way analysis of variance (ANOVA) (groups CD, CD + ATV_2_, HD, HD + ATV_1_, HD + ATV_2_, HD + ATV_3_) accompanied by the Student-Newman-Keuls test and differences between the groups were determined by the Student's t test (Without CoQ_10_ vs With CoQ_10_) (p < 0.05). ^a^Statistically different from either CD or CD + CoQ_10_ (p < 0.05); ^b^statistically different from either HD or HD + CoQ_10_ (p < 0.05); ^1^statistically different from the same group without CoQ_10_.

## Discussion

HD administered to rats induced an increment in serum cholesterol and triacylglycerols (Figure 1A). These results are consistent with previous studies [9,10]. As expected, when ATV was administered, cholesterol and triacylglycerols showed a dose dependent decrement (p < 0.05). Supplementation with CoQ_10_ increased the effects of ATV on cholesterol levels. Rats with HD showed a slight increased concentration of serum triacylglycerols. The administration of ATV did not reduce these TAG levels, however, all values were minor when CoQ_10_ was supplemented (Figure 1C, D).

In our study it was observed a significant diminution of serum cholesterol in rats that received ATV + CoQ_10_ in comparison with those groups that did not receive CoQ_10_. These results support a better hypolipidemic effect of ATV in the presence of CoQ_10_. This improvement in the effect of ATV by CoQ_10_ has already been reported in Guinea pigs [10,11].

HDL-C showed a significant decrease in the HD group. The administration of ATV to HD rats did not increase HDL-C values but kept them similar to those observed in CD group. However, HDL-C values were higher in groups CD + CoQ_10_, HD + CoQ_10_ and CD + ATV_2_ + CoQ_10_ compared to CD without CoQ_10_ supplementation (Figure 1E, F). These results confirm the beneficial effect of ATV on HDL-C levels and even the more beneficial effect of CoQ_10_ supplementation on at least in groups CD + CoQ_10_, HD + CoQ_10_ and CD + ATV_2_ + CoQ_10_.

It is well known that statins inhibit cholesterol biosynthesis in the liver, decrease the intracellular cholesterol content, augment low density lipoprotein-receptor (LDL-R) synthesis as well as the cholesterol uptake by the liver, and diminish serum total cholesterol concentration [12]. In addition, statins increment HDL-C levels throughout an increase of apoprotein A synthesis in the liver [13] and a reduced activity of cholesterol ester transfer protein (CETP).

Mabuchi et al. [14] reported that co-administration of ATV-CoQ_10_, favored a significant increase of HDL-C in hypercholesterolemic patients. Singh et al. [15] observed an important increment of HDL-C in patients that received CoQ_10_. However, it has been reported no increase in HDL-C in patients that received simvastatin and CoQ_10_. Nevertheless, it is not clear how is that synergistic effect of CoQ_10_ on ATV action. It is well known that CoQ_10_ and cholesterol are synthesized by the same pathway and that high ATV doses produce a significant decrement in CoQ_10_ levels in plasma [14,15] and this decrement in serum CoQ_10_ is related direct or indirectly to the potential liver harm produced by the statin treatment [16]. On the other hand, CoQ_10_ administration may inhibit the expression of the apo A-I receptor, increasing apoprotein A-I and increasing HDL-C levels [15].

Our results show that rats that received atorvastatin (0.2 mg/day) and CoQ_10_ had lower levels of serum glucose than the same group without CoQ_10_ (Figure 1G, H). In addition, CoQ_10_ regulates glucose levels throughout a diminution of oxidative stress [17]. On the other hand, other reports have shown that ATV lowers serum cholesterol, increases glucose blood levels and raises insulin resistance [18]. These data altogether suggest that co-administration of CoQ_10_ and ATV improves glucose metabolism in the hypercholesterolemic state.

Some reports indicate that CoQ_10_ administration improves pancreatic beta cells function, increases insulin sensitivity and preserves the mitochondrial function in the pancreas [19]. Moreover, CoQ_10_ diminishes lipoperoxidation and raises glucose uptake. These results suggest that CoQ_10_ improves glucose metabolism in hypercholesterolemia under atorvastatin treatment.

It was also observed in ATV-treated rats an increment in ALT and AST serum activity. Previous studies have also shown an increase in serum aminotransferases (ALT y AST) in rats that received HD and ATV [20,21]; these results were related to liver damage. In accordance with these results, other animal models with hypercaloric diet are predisposed to hyperlipidemia and liver steatosis [21,22]. On the other hand, a study employing ATV in rats did not show change in the activity of serum aminotransferases [10].

The high serum aminotransferases levels in rats with cholesterol-rich diet are related to liver damage. This harm is due to membrane damage in hepatocytes which produces a lessened antioxidant and detoxification capacity of the liver [21]. Other studies have reported higher activity of transaminases produced by statin administration to rats [21,22]. On the contrary, our study showed a slight decrement of AST and ALT activity in ATV-CoQ_10_, treated animals compared with those that received only ATV. Also, Mabuchi et al. [14] observed a diminution of AST and ALT in patients treated with ATV and CoQ_10_. Moreover, Abbas and Sakr [23] reported a diminution of AST and ALT activity in Guinea pigs that received simvastatin-CoQ_10_, comparing with animals that only received simvastatin. All these results together may assign a protector effect of CoQ_10_ on the hepatocytes of rats fed a cholesterol-rich diet.

In our study, it was observed that ATV lessened cholesterol and triacylglycerol concentration in the liver in a dose-dependent manner in hypercholesterolemic rats. Several reports suggest that this increment induced by HD contributes to the liver steatosis, as well as the dietary fatty acids and cholesterol promote the lipid accumulation in the hepatocytes. These cells have receptors for the transcription factor PPAR-α, allowing fatty acid oxidation in mitochondria, microsomes and peroxisomes [24]. As a result, fatty acids oxidation products (hydrogen peroxide, oxygen superoxide and lipid peroxides) are produced and induce lipid peroxidation and oxidative stress [25].

Several studies have shown that a cholesterol-rich diet given to rats produce a fatty liver, hypertrophy of the liver and macroscopic alterations [25,26] as a consequence of hepatocyte cholesterol saturation; the novo cholesterol synthesis lowers and consequently it is produced a diminished uptake of LDL by its receptors. Results from other studies show a lower activity of HMG-CoA reductase and lower expression of LDL receptors in the liver from rats fed a high fat diet [27]. Our study showed a significant decrease of cholesterol and triacylglycerol levels in the liver of animals treated with ATV and CoQ_10_, compared with those rats that only received ATV. These results are in coincidence with other reports [11] that suggest CoQ_10_ improves the hypolipemiant action of statins. As we already mentioned it is not currently known the mechanism by which CoQ_10_ increases statins action. Some studies suggest CoQ_10_ influences the negative feedback of hepatic cholesterol. Moreover, cholesterol metabolism in the liver is mediated by lanosterol 14α demethylase (CYP51) throughout the sterol regulatory binding proteins (SREBPs) [28,29]. Previous reports studying the effect of the reduced form of CoQ_10_ on the liver cholesterol metabolism, showed an antagonistic action on the ligand binding to X receptor (LXR) [30]. Liver LXRs induce SREBP-1c, a transcription factor that controls the expression of several genes involved in cholesterol biosynthesis and its reverse transport. On the other hand, the amount of dietary cholesterol to be absorbed at the intestine is controlled by a transporter family (ABC), localized at the enterocyte membrane. These proteins pour out cholesterol from the enterocytes to the intestine lumen. The hydroxyl group of the reduced form of CoQ_10_ is important for this antagonistic action on ABC transporter genes throughout the LXR ligand [31]. This mechanism may explain the cholesterol diminution in serum and liver observed in all animals that received ATV and CoQ_10_ in our study.

It is generally accepted that a cholesterol-rich diet produces structural mitochondrial alteration in the liver and higher production of reactive oxygen species (ROS) with hepatocellular damage [31,32]. Electron microscope studies in rats with non-alcoholic fatty liver, show scarce mitochondria, higher in size, deformed, hypodense, with paracrystaline inclusions, hepatosteatosis and altered fatty acid oxidation [33]. In our study HD produced a lower respiratory control. Other authors suggest that a high-lipid diet induce deterioration of complex I (NAD: ubiquinone oxidoreductase) and II (succinate dehydrogenase) of the mitochondrial chain [10]. Other reports suggest that statins like pravastatin lessen the mitochondrial respiratory control affecting complex I and IV (cytochrome c oxidase) in skeletal muscle [33,34]. In addition, simvastatin induces myotube atrophy and cell loss associated with impaired ADP-stimulated maximal mitochondrial respiratory capacity, mitochondrial oxidative stress [35]. It is known that ATV reduces the cholesterol-phospholipid ratio in cellular membrane, raising its fluidity and the activity of ATPase Na^+^/K^+^[36]. All these modifications in cellular membrane affect the activity of participating enzymes of the mitochondrial electron transport chain with probable alteration of its bioenergetic function. Our results support that the mitochondrial respiration diminution observed in animals treated with ATV can be attributed to lower levels of CoQ_10_.

The mitochondrial respiratory chain and particularly complex I and complex III (ubiquinone:cytochrome c oxidoreductase) are able to produce an anion superoxide from oxygen. In hepatocytes from normal rats this is a tenuous production that doesn’t interfere with the respiratory chain activity, but is functioning as a mitochondrial protective antioxidant system. On the other hand, CoQ_10_ is an invaluable component of the mitochondrial respiratory chain [36,37] and a diminution in its availability affects for sure the energetic metabolism. It is known that the administration of CoQ_10_ and simvastatin increased the activity of complex I in cardiomyocytes but decreased with simvastatin alone. On the other hand, there are evidences suggesting that the significant decrease in ATP concentration in simvastatin-treated rats was due to CoQ_10_ deficiency [38]. Our results show a higher mitochondrial RC in all groups that received ATV and CoQ_10_. On the same way, Kimura et al. [39] communicated the increase in muscle fibers contraction in rats that received CoQ_10_ due to improvement of cell membrane. A study suggests CoQ_10_ may reduce symptoms related to heart failure and increased energy production in heart muscle [40]. Statins sometimes cause muscle pain and oral CoQ_10_ might reduce this pain [40,41].

In our study we observed that ATV decreased mitochondrial respiration but ATV and CoQ_10_ improved mitochondrial function using succinate as substrate. Statins have been associated with a reduction in serum and muscle tissue coenzyme Q_10_ levels that may play a role in statin-induced myopathy. Aged people appear to be more susceptible to coenzyme Q_10_ deficiency. Athletes also require the most efficient oxygen consumption by mitochondria for their performance, and are more susceptible to CoQ_10_ deficiency. However, there is not a general opinion regarding the effectiveness of CoQ_10_ supplementation. It seems that those that would gain the major benefit from this supplementation are the hypercholesterolemic patients.

## Conclusions

Our results support that the combination ATV and CoQ_10_ improves biochemical parameters, liver mitochondrial respiratory function in hypercholesterolemic rats with high ATV doses. These results have implications when considering statin safety and effectiveness. Supplementation with CoQ_10_ may add beneficial effects in hypercholesterolemic patients, being harmless for human beings and also having a hepato-protector action.

## Methods

### Chemical compounds

All chemicals were purchased from Sigma (St Louis, Mo, USA): sucrose, HEPES (4 - (2-hydroxiethyl)-1-piperazine ethane sulfonic acid, EGTA (ethylen glycol tetraacetic acid), succinic acid, phosphoric acid, magnesium chloride, potassium chloride, bovine serum albumin, sodium deoxycholate, cholesterol, adenosine 5'-diphosphate sodium salt (ADP), ethanol, coenzyme Q_10_, and Atorvastatin (Lipitor) from Pfizer.

### Animals and diets

Male Wistar rats were obtained from Unidad de Producción, Cuidado y Experimentación Animal (UPCEA), División Académica de Ciencias de la Salud (DACS), Universidad Juárez Autónoma de Tabasco (UJAT), verified by the Secretaria de Agricultura, Ganadería y Recursos Pecuarios (SAGARPA 2005). All procedures were subject to regulations of animal experimentation from the Norma Oficial Mexicana NOM-062-ZOO-1999, and the International Guide for caring and use of laboratory animals NRC 2002, with the approval of the Ethics Committee of Faculty of Medicine, UNAM (PAPIIT-IN221914). Rats at the age of 7 weeks and 180–200 g body weight were maintained under controlled housing conditions: 55% humidity, 21 ± 1°C temperature, 12–12 h light–dark cycle. The Harlan Laboratories diet (2018S) with 18.6% protein, 44.2% carbohydrates, 6.2% fat was used to prepare the diets of each groups (n = 8 each one). The desing of this study included two experiments:

Experiment 1: The total number of animals used was 48, divided into 8 groups. No one had CoQ_10_ supplementation:

CD Control diet

CD + ATV_2_ (atorvastatin 0.56 mg/day)

HD Hypercholesterolemic diet (2% cholesterol, 0.6% sodium deoxycholate).

HD + ATV_1_ (atorvastatin 0.2 mg/day)

HD + ATV_2_ (atorvastatin 0.56 mg/day)

HD + ATV_3_ (atorvastatin 1.42 mg/day)

Experiment 2: The total number of animals used was 48, divided into 8 groups. All groups received CoQ_10_ 0.57 mg/day as supplement:

CD Control diet

CD + ATV_2_ (atorvastatin 0.56 mg/day)

HD Hypercholesterolemic diet (2% cholesterol, 0.6% sodium deoxycholate).

HD + ATV_1_ (atorvastatin 0.2 mg/day)

HD + ATV_2_ (atorvastatin 0.56 mg/day)

HD + ATV_3_ (atorvastatin 1.42 mg/day)

All Animals were given free access to water and diets during the six week experimental time. Diets were freshly prepared each day with grinded food. Body weight was assessed once a week. All animals were kept under the above mentioned experimental conditions for a 6-week period. At the end of treatment and after a 12 h food withdrawal, rats were sacrificed by decapitation. The liver was removed, weighted and 0.5 g were used for biochemical determinations, the remaining liver tissue was used for the assay of mitochondrial respiratory function.

### Biochemical parameters

Blood was collected and serum was immediately frozen and stored at −70°C until the biochemical determinations were performed. Serum levels of glucose, cholesterol, triacylglycerols, high-density lipoprotein-cholesterol (HDL-C), aspartate aminotransferase (AST), and alanine aminotransferase (ALT) were analyzed using a Clinical Chemistry System from Random Access Diagnostics.

### Cholesterol and triacylglycerols from the liver

Liver lipids were extracted according to the Folch et al. [42] (1957) procedure, whereas triacylglycerols and cholesterol concentrations were measured using enzymatic colorimetric determinations according to diagnostic kits from BioSystems Laboratories.

### Mitochondria isolation

Hepatic mitochondria were harvested by centrifugation, washed twice with 250 mM sucrose, 0.5 mM HEPES, 0.5 mM EGTA (SHE) buffer and resuspended in SHE pH 7.2 at a final ratio of 5 ml/g wet weight. Subsequent steps were carried out in the same buffer at 4°C and mitochondria were isolated by differential centrifugation. Briefly, cell debris was eliminated by centrifugation at 3000 g for 10 min, the mitochondrial pellet was obtained by spinning the supernatant for 10 min at 12000 g, it was washed once to eliminate cytosolic contamination, and suspended with SHE buffer to a final protein concentration of 10–30 mg/mL. Protein determination was performed using the Bradford method [43] (1976).

### Oxygen consumption

Respiratory measurements were carried out in 3.5 ml of air-saturated medium with 5 mM succinate, 2 mM MgCl_2_, 2 mM H_3_PO_4_, 2 mM EGTA, 30 mM HEPES, 0.1% BSA, pH 7.2 at 24°C. Oxygen consumption was determined using a Clark-type oxygen electrode. Data are expressed as the respiratory control ratio (RCR), which is a relative value of state 3 and state 4 that indicates the respiratory coupling in availability of ADP [44] (1967).

### Statistical analysis

Comparisons between means were performed using One-way analysis of variance (ANOVA), followed by Student–Newman–Keuls test and differences between the groups were determined by the Student's t test (without CoQ_10_ vs with CoQ_10_). Differences were considered to reach statistical significance when p < 0.05. For the *post hot* calculation of the statistical power in the ANOVA test for the experiment, we used the G*Power 3.0.10 software (Franz Faul, Universität Kiel, Germany). We used 20% difference in group (i.e. effect size), α level of 0.05, Total sample size: 96 and number of groups 8, and the value of 16 animals needed, then the power was of 1.00.

## Abbreviations

CoQ10: Coenzyme Q_10_; CD: Control diet; HD: Cholesterol-rich diet; ATV: Atorvastatin; RC: Respiratory control; RCR: Respiratory control ratio; TAG: Triacylglycerols; TC: Total cholesterol; HDL-C: High-density lipoprotein-cholesterol; AST: Aspartate aminotransferase; ALT: Alanine aminotransferase; ATP: Adenosinetriphosphate; ADP: Adenosinediphosphate; ANOVA: Analysis of variance.

## Competing interests

The authors declare that they have no competing interests.

## Authors’ contributions

JCDZ and IEJR contributed the design and conducted the study, collection, analysis, interpretation of data and writing of the manuscript. AJS and MTEG carried out biochemistry analysis and respiratory measurements. DYBO participated in data collection and data analysis. TRF contributed in acquisition of funding and critically revising the manuscript. CATZ and MAJO performed statistical analyses and manuscript preparation. All authors read and approved the final manuscript.

## Authors’ information

^1^Carretera Rancho, Sur 4a Sección. C.P. 86650. Comalcalco, Tabasco, México.

^2^Av. Gregorio Méndez Magaña. No. 2838-A. Col. Tamulté. C.P. 86300, Villahermosa, Tabasco, México.

^3^Av. Universidad 3000. Ciudad Universitaria, Coyoacán, 04510. México, D.F., México.
